# EFFECTS OF PROBIOTICS SUPPLEMENTATION ON SKIN WOUND HEALING IN DIABETIC RATS

**DOI:** 10.1590/0102-672020190001e1498

**Published:** 2020-07-08

**Authors:** Letícia Fuganti CAMPOS, Eliane TAGLIARI, Thais Andrade Costa CASAGRANDE, Lúcia de NORONHA, Antônio Carlos L. CAMPOS, Jorge Eduardo F MATIAS

**Affiliations:** 1 Postgraduate Program in Surgical Clinic, Federal University of Paraná, Curitiba PR, Brazil; 2 Professional Master Program in Industrial Biotechnology, Positivo University, Curitiba, PR, Brazil; 3 Laboratory of Experimental Pathology, Pontifical Catholic University of Paraná, Curitiba, PR, Brazil

**Keywords:** Probiotics, Diabetes mellitus, Wound healing, Alloxan, Probióticos, Diabete melito, Cicatrização, Aloxano

## Abstract

**Background:**

Chronic wounds in patients with Diabetes Mellitus often become incurable due to prolonged and excessive production of inflammatory cytokines. The use of probiotics modifies the intestinal microbiota and modulates inflammatory reactions.

**Aim:**

To evaluate the influence of perioperative supplementation with probiotics in the cutaneous healing process in diabetic rats.

**Methods:**

Forty-six rats were divided into four groups (C3, P3, C10, P10) according to the treatment (P=probiotic or C=control, both orally administered) and day of euthanasia, 3^rd^ or 10^th^ postoperative days. All rats were induced to Diabetes Mellitus 72 h before starting the experiment with alloxan. Supplementation was initiated five days before the incision and maintained until euthanasia. Scalpel incision was guided by a 2x2 cm mold and the wounds were left to heal per second-intention. The wounds were digitally measured. Collagen densitometry was done with Picrosirius Red staining. Histological parameters were analyzed by staining by H&E.

**Results:**

The contraction of the wound was faster in the P10 group which resulted in a smaller scar area (p=0.011). There was an increase in type I collagen deposition from the 3^rd^ to the 10^th^ postoperative day in the probiotic groups (p=0.016), which did not occur in the control group (p=0.487). The histological analysis showed a better degree of healing in the P10 group (p=0.005), with fewer polymorphonuclear (p<0.001) and more neovessels (p=0.001).

**Conclusions:**

Perioperative supplementation of probiotics stimulates skin wound healing in diabetic rats, possibly due to attenuation of the inflammatory response and increased neovascularization and type I collagen deposition.

## Graphical Abstract

[Fig f1a]

**Figure f1a:**
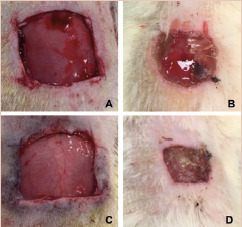
Example of wound contraction area at: A) day of surgery in the control group; B) 7PO in the control group; C) day of surgery in the probiotic group; D) 7PO in probiotic group

## RESUMO

**Racional:**

Feridas crônicas em pacientes diabéticos muitas vezes se tornam incuráveis devido à produção prolongada e excessiva de citocinas inflamatórias. A utilização de probióticos modifica a microbiota intestinal e modula reações inflamatórias.

**Objetivo:**

Avaliar a influência da suplementação perioperatória com probióticos no processo de cicatrização cutânea em ratos diabéticos.

**Método:**

Quarenta e seis ratos foram divididos em quatro grupos (C3, P3, C10, P10) conforme tratamento (P=probiótico ou C=controle, via oral) e dia de eutanásia: 3^o^ ou 10^o^ dia de pós-operatório. Todos os ratos foram induzidos ao diabete melito 72 h antes de iniciar o experimento com aloxana. A suplementação foi iniciada cinco dias antes da operação e mantida até a eutanásia. Foi realizada incisão com bisturi guiada por molde de 2x2 cm e a ferida foi deixada para cicatrizar por segunda intenção. As feridas foram medidas digitalmente. A densitometria de colágeno foi determinada com coloração picrosirius red. A histologia foi avaliada por coloração com H&E.

**Resultados:**

A contração da ferida foi maior no grupo P10, o que resultou em menor área cruenta (p=0,011). Houve aumento do colágeno tipo I do 3^o^ para o 10^o^ dia de pós-operatório no grupo P10 (p=0,016), o que não ocorreu no grupo controle (p=0,487). A análise histológica mostrou melhor grau de cicatrização no grupo P10 (p=0,005), com menos polimorfonucleares (p<0,001) e mais neovasos (p=0,001).

**Conclusões:**

A suplementação perioperatória de probióticos promove aceleração da cicatrização cutânea em ratos diabéticos, possivelmente por atenuar a resposta inflamatória e aumentar a neovascularização e a deposição de colágeno tipo I.

## Graphical Abstract

[Fig f1as]

**Figure f1as:**
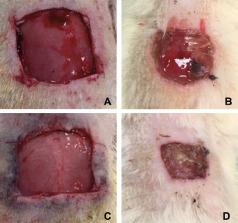
Exemplo da diferença de área de contração da ferida: A) grupo controle no dia da operação; B) 7PO no grupo controle; C) grupo probiótico no dia da operação; D) 7PO no grupo probiótico

## INTRODUCTION

Approximately 425 million adults are affected by Diabetes Mellitus (DM) worldwide, and this disease was responsible for four million deaths in 2017 and a US$ 727 billion spending on health care in the United States (12% of total healthcare spending with adults)^10^.

Diabetic patients present impairment in healing due to imbalance of the inflammatory response, with prolonged accumulation of inflammatory cells and excessive production of inflammatory cytokines, alteration of collagen synthesis and reduction of tensile strength^6^ ^,^ ^7^ ^,^ ^14^ ^,^ ^19^ ^,^ ^22^. These factors when associated result in reduced wound resistance and therefore increased dehiscence which, added to the larger risk of infections in this group, result in increased hospitalization time and mortality rate^3^ ^,^ ^6^ ^,^ ^7^ ^,^ ^14^ ^,^ ^22^.

The combination of impairment in the healing process with peripheral vascular disease and the difficulty in the perception because of the neuropathy in extremity injuries result in an increased risk for the development of diabetic foot disease. Diabetic foot ulcer is the leading cause of hospitalization of DM patients in developed countries and is among the leading causes of morbidity and mortality, with an average healing time of six months and need for amputation in up to one third of the cases. This complication still demands studies addressing the complexity of its management^3^ ^,^ ^6^ ^,^ ^7^ ^,^ ^14^. 

Damage to the epithelial barrier results in dermis rupture and epithelial cell stress, requiring repair through healing. Skin healing is characterized by a dynamic process that involves a complex network of extracellular interactions, chemical mediators, and inflammatory cells. The main objective of this process is the restoration of tissue integrity and the maintenance of homeostasis. The healing process involves three sequential phases, which might be subdivided in four or five phases, that are dynamically related and not individualized: inflammatory phase, cellular proliferation phase, conjunctive tissue formation phase, contraction phase and the final wound remodelling phase^2^ ^,^ ^21^ ^,^ ^24^.

Chronic wounds in patients with DM often do not follow this orderly progression and may persist in the inflammatory phase and do not progress to the next stages of wound healing. The transition from inflammatory to proliferative stage in wound healing is the subject of intensive current research, and systemic regulation of inflammation plays an important role at this stage^4^ ^,^ ^7^ ^,^ ^11^ ^,^ ^14^ ^,^ ^19^.

The relationship between the healing process and the interaction with the skin microbiota is already well established. It has been shown that the local microbiota may provide tonic stimulation to the host´s immune system and prevent invasion of other pathogenic microbes. Loss of microbial diversity often results in prolonged inflammation and delay of wound healing process^25^ ^,^ ^28^. Recent studies suggest that, in addition to interaction with the local microbiota, changes in the intestinal microbiota may also positively or negatively affect the wound healing process by producing antimicrobial molecules, and regulating the immune and inflammatory response^1^ ^,^ ^11^ ^,^ ^12^ ^,^ ^26^ ^,^ ^28^. Arck et al^1^ proposed the existence of a “brain-intestine-skin axis”. According to this theory, intestinal bacteria may interfere with remote skin healing by modulating release of inflammatory cytokine expression. 

Therefore, modulating the intestinal microflora might be an important strategy for improving the skin healing process. The tools for modulation of intestinal microbiota are prebiotics, probiotics, symbiotics and stool transplantation, in addition to dietary changes. Commercial probiotics products contain known and quantified bacteria, and the strains most applicable in clinical situations are lactobacilli (*Lactobacillus casei, Lactobacillus acidophilus, Lactobacillus rhamnosus*) and bifidobacterium (*Bifidobacterium lactis*)^8^. The main link between probiotic use and healing is modulation of inflammation, which can have positive effects on tissue repair.

The objective of this study was to evaluate the influence of perioperative probiotic supplementation on the skin healing process of diabetic rats.

## METHODS

### Experimental model

The study was part of the Research on Tissue Healing Group of the Graduate Program in Surgery of the Federal University of Parana, Curitiba,PR, Brazil. The project was conducted according to the rules provided by Federal Law No. 11.794, of October 8, 2008, standards provided by the National Council for Animal Experimentation Control. The study protocol was approved by the Animal Use Ethics Commission of the Positivo University, where the experiment was carried out. A total of 46 males adult Wistar rats (*Rattus norvegicus albinus*, Rodentia Mammalia) were used. The rats were divided into two groups: probiotics (P) that received Probiatop^®^, and control (C), which received maltodextrin. Both supplementations were orally administrated with the aid of a spatula, at a dose of 250 mg probiotic or maltodextrin once a day. Each group was subdivided into two subgroups according to the day of euthanasia: 3^rd^ or 10^th^ postoperative (PO, subgroups P3=12 rats, C3=12 rats, P10=11, C10=11 rats). During the experimental period the rats were kept under controlled temperature (22±1° C) and 12 h dark/light cycles, with water and rat chow Presence^®^ (Purina, São Paulo, Brazil) ad libitium. The groups received probiotic or maltodextrin for five days before the creation of the skin excisional wound and maintained this consumption until the day of euthanasia. The probiotic offered was Probiatop^®^, composed of four strains (doses 1x10^9^ CFU/g) *Lactobacillus paracasei* LPC-37^®^, *Bifidobacterium lactis* HN0019^®^, *Lactobacillus rhamnosus* HN001^®^ and *Lactobacillus acidophilus* NCFM^®^. These doses are within the evidence-based recommendations for humans^18^. The rats were weighed before the DM induction, on the day of surgery and on days 3, 7 and 10 postoperatively, with an electronic scale (AM 5500^®^ Marte, São Paulo, SP, Brasil).

### Induction of diabetes mellitus

All rats were induced to DM 72 h before starting the preoperative probiotic or placebo supplementation (eight days before surgery). After 16 h of fasting, the rats underwent isoflurane inhalation anesthesia in a glass bell and were then placed in a decubitus position to receive injection of alloxan monohydrate (*Sigma*Chemical Co, USA) through the caudal vein. Alloxan was diluted in distilled water and applied in a single dose of 40 mg/kg^16^. After 1h 30 min rat chow and water were reintroduced. The confirmation of the diagnosis of DM was done 48 h after induction, and fasting glucose >200 mg/dl was standardized for diagnosis. Animals that died on post induction period were replaced to avoid compromising the final group numbers. Glucose levels were assessed by taking manually a drop of blood from the tail, which were placed on reagent strip and evaluated by the One Touch Select Simple^®^ glucometer (Johnson&Johnson, Brazil). Blood glucose levels were evaluated on days 0, 3, 7 and 10 postoperatively.

### Surgical procedure

On the day of surgery, the rats were anesthetized by inhalation of isoflurane in a glass bell and were premedicated with 4 mg/kg intramuscularly morphine. Dorsal region trichotomy and antisepsis were performed, followed by a scalpel incision guided by a 2x2 cm square mold, resecting the entire thickness of the skin and exposing the dorsal muscular fascia. The wounds were left open to heal by second intention. Postoperative analgesia was performed with oral acetominophen at a daily dose of 100 mg/kg orally, diluted in the water, until the 4^th^ postoperative day.

### Wound contraction assessment

Wounds were macroscopically evaluated by digital photographs taken on days 1, 3, 7, and 10. For the analysis of wound contraction rates the wound area was measured using the Image-Pro Plus^®^ 4.5 software (Media Cybernetics, Rockville, Maryland, USA). The wounds were photographed at a standard distance of 15 cm.

### Euthanasia

On the 3^rd^ (groups C3 and P3) and 10^th^ day (groups C10 and P10) postoperatively the rats were euthanized in a closed glass bell system with isoflurane. Immediately after death, the wound was excised with a 1 cm margin.

### Collagen densitometry

Collagen densitometry was performed to identify and quantify type I and type III collagen using Picrosirius Red F3BA (PSR) staining, polarized light optical microscopy, and software image analysis. Images were recorded by AxioVision 4.9 Software (Zeiss, Germany) and analysed by Image-Pro Plus^®^ 4.5 software (Media Cybernetics, Rockville, Maryland, USA).

### Histological study by H&E staining

The samples were cut into rotary microtome blocks, with five micrometer thick sections, and subjected to H&E staining. Then, the pieces were submitted to the dehydration and diaphanization processes in xylol, and stained with H&E. Slide reading was performed using an Olympus BX40 (Tokyo, Japan) optical microscope with 20x magnification. The histological analysis included the types and number of predominant cells of inflammatory reaction (polymorphonuclear), presence of interstitial edema and vascular congestion, and the degree of fibroblast, neovessels and monocyte tissue formation. These data were classified as accentuated (3), moderate (2) and discrete (1) and transformed into quantitative variables by assigning the index to histological findings. The presence of polymorphonuclear, edema and congestion were indicative of acute inflammatory process, punctuating negatively, and the formation of fibroblasts, neovessels and monocytes were indicative of chronic inflammatory process, punctuating positively. After the indices were assigned, they were summed to total the final score for further statistical evaluation among the studied groups^27^.

### Statistical analysis

Results were described as mean and standard deviation (SD). For comparison between groups, the nonparametric Mann-Whitney test was used. Comparisons between assessment days within the same group were made using the non-parametric Kruskal-Wallis test. For correlation analysis, the Spearman coefficient was used. Values ​​of p<0.05 indicated statistical significance. Data were analysed using the IBM SPSS Statistics^®^ software, v.20.

## RESULTS

Groups C3 and C10 showed weight loss between the induction of DM and the 10^th^ day (p<0.001), while groups P3 and P10 showed no weight reduction within the same period (P3=0.789, P10=0.433). 

Rats receiving probiotics had lower blood glucose levels at the time of surgery for group P10 as compared with C10 (321±146 vs*.* 541.2±112 mg/dl, p=0.001), as well as at the 3^rd^ PO (281±132 vs. 405±147 mg/dl, p=0.040). 

**FIGURE 1:**
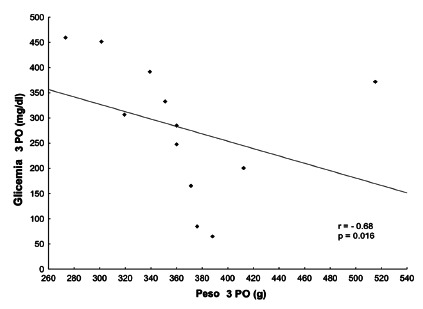
Spearman correlation coefficient for blood glucose and body weight on 3^rd^ PO day for the control group (C3)

The glycemia of the 3^rd^ PO in group C3 was negatively correlated with the weight of the 3^rd^ PO in this group (Spearman correlation coefficient= -0.68, p=0.016), indicating that the higher the blood glucose values, the lower the weight (Figure 1). There was no significant difference on the other days.

**FIGURE 2:**
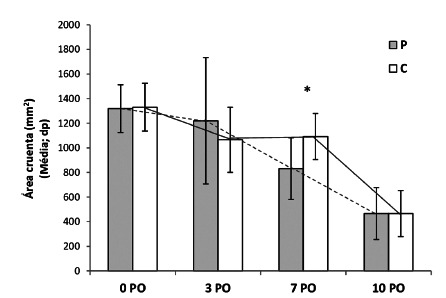
Wound contraction in both control and probiotic rats. Note that the use of probiotic accelerated wound healing. This difference was significant on day 7^th^

**FIGURE 3:**
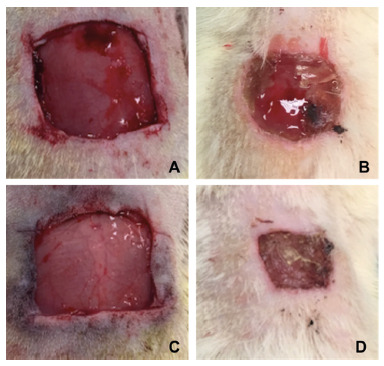
Example of wound contraction area at: A) day of surgery in the control group; B) 7PO in the control group; C) day of surgery in the probiotic group; D) 7PO in probiotic group

Wound contraction was greater in group P10 as compared to group C10, which resulted in smaller wound area in the 7^th^ PO (847±189 vs. 1054±269 mm^2^, p=0.049, Figures 2 and 3).

*p=0,011

In the control groups there was a positive correction between weight and wound area according to the Spearman’s Correlation Coefficient (C3=0.69, p=0.014; C10=0.63, p=0.037).

**FIGURE 4:**
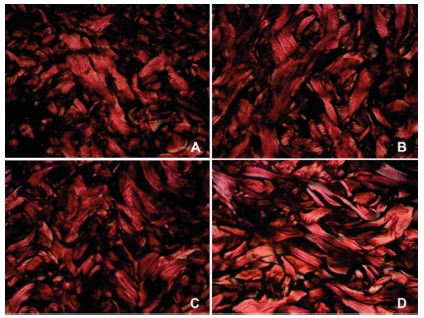
Example of type I collagen deposition: A) 3^rd^ PO on control group; B) 10^th^ PO on control group; C) 3^rd^ PO on probiotic group; D) 10^th^ PO on probiotic group

There was an increase in type I collagen deposition from the 3^rd^ to the 10^th^ day in the groups receiving probiotics (p=0.016), which did not occur in the control groups (p=0.487, Figure 4). There was no significant difference for collagen type III analysis.

**FIGURE 5:**
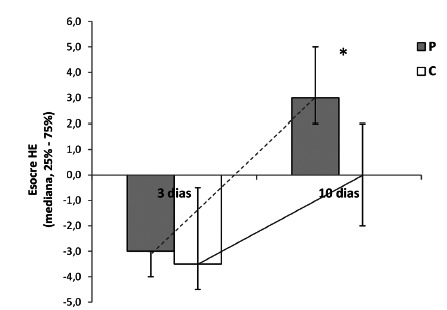
Histological score by H&E to evaluate degree of wound healing process on control (C) and probiotics (P)

**FIGURE 6:**
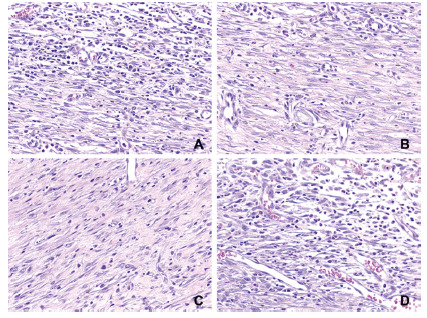
Example of microphotographies demonstrating: A) accentuated presence of polymorphonuclear cells on control group; B) discrete presence of polymorphonuclear cells on probiotic group; C) discrete presence of neovessel on control group; D) accentuated presence of neovessels on probiotic group (20x)

Analysis of the final histological score by H&E showed a better healing process in the P10 group when compared to C10 (P10=3 vs. C10=0, p=0.005, Figure 5), with less polymorphonuclear cells (p<0.001) and more neovessels (p=0.001, Figure 6). There were no significant difference for the other parameters.

*p=0.005

## DISCUSSION

The present study, in diabetic rats, demonstrated benefit of oral probiotics supplementation in wound healing, mature collagen deposition, neovascularization stimulation and reduction of the inflammatory process, as well as attenuating weight loss and improving glycemic control. Some previous experimental studies have also shown benefits of the use of prebiotic or probiotic on wound healing. Huseini et al^9^ showed that the use of Kefir products was able to improve wound healing in rats. Importantly, however, none of these studies included diabetic rats.

In addition, patients’ chronic leg ulcer cells collected after topical treatment with *Lactobacillus plantarum* for 10 days showed lower bacterial load on the wound, in addition to inducing wound healing^20^. 

In the present study, wound contraction was faster in the probiotic group compared to control, resulting in a smaller wound area, due to increased type I collagen deposition and increased of neovessels formation. A recent study^18^ showed similar results with 12-week probiotic supplementation *Lactobacillus acidophilus*, *Lactobacillus casei*, *Lactobacillus fermentum* and *Bifidobacterium bifidum* (2×10^9^ UFC/g each) in diabetic patients with diabetic foot ulcer. In that study, probiotics supplementation reduced the ulcer size and resulted in better glycemic control. The anti-infectious mechanisms of the probiotics in patients with diabetic foot ulcers suggested on that study included the improved ability to fight with pathogenic microorganisms or by modulating host immune responses, the production of various antimicrobials substances and their anti-inflammatory properties^18^.

The healing remodeling phase is characterized by organized collagen deposition, with replacement of the initial collagen (type III) and production of thicker and more organized mature collagen (type I), approaching the composition of the healthy dermis^2^. In the present study it was possible to observe an increase of mature collagen deposition (type I) in the group that received probiotics as compared to controls.

Another finding of this study was the higher neovascular formation in group P10 compared to C10. The number of vessels have been shown to be decreased after DM induction on rats^19^. Improvement of neovascularization via angiogenesis is essential for the proper reepithelialization process by ensuring adequate nutrient supply, immune cells and oxygen. Rapid and robust vascular growth creates a vascular bed with more capillaries than the normal tissue^4^ ^,^ ^5^ ^,^ ^18^ ^,^ ^21^ ^,^ ^24^.

The reduction of polymorphonuclear cells in group P10 as compared to C10 suggests that perioperative supplementation with probiotics was able to attenuate prolonged and excessive inflammatory cytokine expression. Neutrophils infiltrate shortly after wound injury for microbial sterilization and removal of foreign compounds, and then regression should occur until production ceases when the inflammatory phase is terminated. If neutrophils persist in the lesion tissues, there may be excessive production of inflammatory cytokines, which makes the scar refractory. Skin biopsy analysis in a previous study showed that diabetic patients had higher immune cell infiltration when compared to healthy individuals, and that this increased inflammation is associated with failure to heal in patients with DM. The use of topical probiotic (*Lactobacillus plantarum*) in diabetic and non-diabetic patients with chronic infected leg ulcers was able to reduce neutrophils, reduce bacterial load and induce wound healing^20^.

Perioperative probiotic supplementation in diabetic rats prevented weight loss, which occurred in the control group. Insulin is the best known and essential anabolic hormone for maintaining glucose homeostasis and cell growth and differentiation, stimulating liver and adipocyte lipogenesis, protein synthesis and inhibiting degradation. The inability of the body to utilize glucose because of the lack of insulin can lead to catabolism, with proteolysis and lipolysis as mechanisms to provide energy. In this study, the Spearman correlation coefficient indicated that the higher the glycemic value, the lower the body weight, which suggests that perioperative supplementation with probiotics favoured glycemic control and consequently avoided catabolism and weight loss. A recent systematic review with DM2 patients showed that the use of probiotics reduced fasting blood glucose (in 19 studies), glycated haemoglobin (in 13 studies), insulin (in 13 studies) and HOMA-IR (in 10 studies)^13^.

The potential mechanisms of probiotics actions on glycemic control are: 1) direct intraluminal effects on the microbiota, with increase in the production of short-chain fatty acids (SCFAs), intestinal permeability reduction and lipopolysaccharides, and increases the production of glucagon-like peptide 1 (GLP-1); 2) anti-inflammatory and immunomodulatory effects, with reduction of pro-inflammatory cytokines; 3) reduction of oxidative stress, with protective effect of beta cells; 4) effects of gene expression involved in glucose homeostasis and insulin resistance, as sensitization increases via GLUT-4^17^.

The proper treatment of DM can prevent the maintenance of the wound on the inflammation phase and thus promotes the wound healing, by the negative regulation of pro-inflammatory cytokines, the regulation of growth factors, the stimulation of angiogenesis and the epithelialization process^6^ ^,^ ^23^. Malnutrition, especially protein malnutrition, can also impair wound healing by prolonging the inflammatory phase, decreasing fibroblast synthesis and proliferation, angiogenesis and collagen synthesis^2^ ^,^ ^14^. However, it is important to note that in this study there was no correlation between glycemia and wound area and, surprisingly, there was a positive correlation between body weight and wound area, which indicates that the higher the weight, the larger the wound area. Thus, it can be interpreted that the mechanism of action of probiotics in accelerating wound contraction was not related to better glycemic control or weight loss prevention, but rather reducing inflammation.

No experimental study similar to this has been identified so far in the literature. In a clinical study with patients with diabetic foot, similar results were found, without, however, elucidating the involved mechanisms^18^. 

However, the present study has some limitations: the diabetes model used mimics an acute disease condition, due to the Aloxana toxic effect on beta cells^16^, different from the chronic one presented by patients with chronic wound complications. Chronic DM causes numerous complications to the healing process, especially peripheral vascular disease, which were not extrapolated to the rats of this study. Still, the animals in this study were not being treated for DM, and recent studies have been correlating chronic metformin use with changes in microbiota^13^, and were not consuming artificial sweeteners, widely used in diabetes patients and also known as harmful to the gut microbiota^15^. Nevertheless, the beneficial effect of probiotics on the modulation of the inflammatory response was confirmed in this model. Rat microbiota differs from human microbiota and certainly clinical studies investigating the influence of probiotics on wound healing in diabetic patients are needed to define the real benefit as well as to define the optimal dose, perioperative supplementation time and most indicated strains.

## CONCLUSIONS

Perioperative supplementation of probiotics promotes accelerated skin healing in diabetic rats, possibly because their use was associated with attenuation of the inflammatory response, and were also associated with increased neovascularization and increased type I collagen deposition. Probiotics supplementation also prevent weight loss and promote better glycemic control as compared with rats that did not receive probiotics.

## References

[1] Arck P., Handjiski B., Hagen E., Pincus M (2010). Is There a ‘Gut-Brain-Skin Axis’. Experimental Dermatology.

[2] Campos A. C. L., Borges-Branco A., Groth A. K (2007). Cicatrização de Feridas. ABCD Arq Bras Cir Dig (São Paulo).

[3] Carro G. V., Saurral F. S., Witman E. L (2018). Diabetic Foot among Hospitalized Patients in Latin
America. Medicina.

[4] Dinh T., Tecilazich A., Doupis J. (2012). Mechanisms Involved in the Development and Healing of Diabetic
Foot Ulceration. Diabetes.

[5] Dipietro L. A (2016). Angiogenesis and Wound Repair: When Enough is
Enough. Journal of Leukocyte Biology.

[6] El-Bahy A. A. Z., Aboulmagd Y. M., Zaki M (2018). Diabetex: A Novel Approach for Diabetic Wound
Healing. Life Sci.

[7] Falanga V (2005). Wound Healing and Its Impairment in the Diabetic
Foot. Lancet.

[8] Flesch A. G. T., Poziomyck A. K., Damin D. D. C (2014). O uso terapêutico dos simbióticos. ABCD Arq Bras Cir Dig.

[9] Huseini H.F., Rahimzadeh G., Fazeli M. R., Mehrazma M., Salehi M (2012). Evaluation of Wound Healing Activities of Kefir
Products. Burns : Journal of the International Society for Burn Injuries.

[10] International Diabetes Federation Https://Www.Idf.Org/Aboutdiabetes/What-Is-Diabetes/Facts-Figures.Html.

[11] Johnson T. R., Gómez B.I., Mcintyre M. K., Dubick M. A. (2018). The Cutaneous Microbiome and Wounds: New Molecular Targets to
Promote Wound Healing. International Journal of Molecular Sciences.

[12] Kiritsi D., Nyström A (2018). The Role of TGFβ in Wound Healing Pathologies. Mechanisms of Ageing and Development.

[13] Koutnikova H., Genser B., Monteiro-Sepulveda M., Faurie J. C. (2019). Impact of Bacterial Probiotics on Obesity, Diabetes and
Non-Alcoholic Fatty Liver Disease Related Variables: A Systematic Review and
Meta-Analysis of Randomised Controlled Trials. BMJ Open.

[14] Lepäntalo M., Apelqvist J., Setacci C., Ricco J. B. (2011). Chapter V: Diabetic Foot. European Journal of Vascular and Endovascular Surgery.

[15] Lobach A. R., Roberts A., Rowland I. R (2019). Assessing the in Vivo Data on Low/No-Calorie Sweeteners and the
Gut Microbiota. Food and Chemical Toxicology.

[16] Lucchesi A. N., Cassettari L. L., Spadella C. T (2015). Alloxan-Induced Diabetes Causes Morphological and Ultrastructural
Changes in Rat Liver That Resemble the Natural History of Chronic Fatty
Liver Disease in Humans. Journal of Diabetes Research.

[17] Miraghajani M., Dehsoukhteh S. S., Rafie N., Hamedani S. G (2017). Potential Mechanisms Linking Probiotics to Diabetes: A Narrative
Review of the Literature. Sao Paulo Medical Journal.

[18] Mohseni S., Bayani M., Bahmani F., Tajabadi-Ebrahimi M (2018). The Beneficial Effects of Probiotic Administration on Wound
Healing and Metabolic Status in Patients with Diabetic Foot Ulcer: A
Randomized, Double-Blind, Placebo-Controlled Trial. Diabetes/Metabolism Research and Reviews.

[19] Oviedo-Socarrás T., Vasconcelos A. C., Barbosa I. X, Pereira N. B (2014). Diabetes Alters Inflammation, Angiogenesis, and Fibrogenesis in
Intraperitoneal Implants in Rats. Microvascular Research.

[20] Peral M. C., Rachid M. M., Gobbato N. M., Martinez M. A. H., Valdez J. C (2010). Interleukin-8 Production by Polymorphonuclear Leukocytes from
Patients with Chronic Infected Leg Ulcers Treated with Lactobacillus
Plantarum. Clinical Microbiology and Infection.

[21] Reinke J. M., Sorg H (2012). Wound Repair and Regeneration. European Surgical Research.

[22] Rosado P., Hsu-Tang C., Chao-Min W., Fu-Chan W (2015). Influence of Diabetes Mellitus on Postoperative Complications and
Failure in Head and Neck Free Flap Reconstruction: A Systematic Review and
Meta-Analysis. Head & Neck.

[23] Salazar J. J., William J. E., Timothy J. K (2016). Diabetes Medications: Impact on Inflammation and Wound
Healing. Journal of Diabetes and Its Complications.

[24] Salgado F. L., Artigiani-Neto R., Lopes-Filho G. J (2016). Growth factors and COX2 in Wound Healing: An Experimental Study
with Ehrlich Tumors. ABCD Arq Bras Cir Dig (São Paulo).

[25] Sorg H., Tilkorn D. J., Hager H., Hauser J., Mirastschijski U (2017). Skin Wound Healing: An Update on the Current Knowledge and
Concepts. European Surgical Research.

[26] Tsiouris C. G., Kelesi M., Vasilopoulos G., Kalemikerakis I., Papageorgiou E. G (2017). The Efficacy of Probiotics as Pharmacological Treatment of
Cutaneous Wounds: Meta-Analysis of Animal Studies. European Journal of Pharmaceutical Sciences.

[27] Vizzotto A. O., Noronha L., Scheffel D. L. H., Campos A. C. L (2003). Influência da cisplatina administrada no pré e no pós-operatório
sobre a cicatrização de anastomoses colônicas em ratos. J Bras Patol e Med Lab.

[28] Wagner N.R.F., Zaparolli M.R., Crus M.R.R., Schieferdecker M.E.M., Campos A.C.L (2018). Postoperative changes in intestinal microbiota and use of
probiotics in roux-en-y gastric bypass and sleeve vertical gastrectomy: an
integrative review. ABCD Arq Bras Cir Dig.

[29] Zheng Y., Ley S. H., Hu F. B. (2018). Global Aetiology and Epidemiology of Type 2 Diabetes Mellitus and
Its Complications. Nature Reviews Endocrinology.

